# Roles of Inflammasomes in Inflammatory Kidney Diseases

**DOI:** 10.1155/2019/2923072

**Published:** 2019-07-21

**Authors:** Jinjin Fan, Kaifeng Xie, Liqin Wang, Nuoyan Zheng, Xueqing Yu

**Affiliations:** ^1^Department of Nephrology, The First Affiliated Hospital, Sun Yat-sen University, Guangzhou, Guangdong Province 510000, China; ^2^Guangdong Medical University, Zhanjiang, Guangdong Province 524001, China; ^3^Department of Radiology, The First Affiliated Hospital, Sun Yat-sen University, Guangzhou, Guangdong Province 510000, China; ^4^Translational Medical Center, The First Affiliated Hospital, Sun Yat-sen University, Guangzhou, Guangdong Province 510000, China

## Abstract

The immune system has a central role in eliminating detrimental factors, by frequently launching inflammatory responses towards pathogen infection and inner danger signal outbreak. Acute and chronic inflammatory responses are critical determinants for consequences of kidney diseases, in which inflammasomes were inevitably involved. Inflammasomes are closely linked to many kidney diseases such as acute kidney injury and chronic kidney diseases. Inflammasomes are macromolecules consisting of multiple proteins, and their formation initiates the cleavage of procaspase-1, resulting in the activation of gasdermin D as well as the maturation and release of interleukin-1*β* and IL-18, leading to pyroptosis. Here, we discuss the mechanism in which inflammasomes occur, as well as their roles in inflammatory kidney diseases, in order to shed light for discovering new therapeutical targets for the prevention and treatment of inflammatory kidney diseases and consequent end-stage renal disease.

## 1. Assembly and Signaling of Inflammasomes

The concept of inflammasome was introduced in 2002 by Dr. Tschopp et al. to describe protein complexes that form within activated immune cells and tissue-resident cells, leading to a series of inflammatory responses including cytokine production and cell death [1, 2]. The inflammasome complex contains three components: inflammasome sensors, adaptors, and effector proteins. The sensor proteins belong to cytosolic pattern recognition receptors (PRRs), which are innate immune sensors capable of recognizing pathogen-associated molecular patterns (PAMPs) and damage-associated molecular patterns (DAMPs) [3]. The known inflammasome sensors include receptors from the NOD-like receptor (NLR) and AIM2-like receptor (ALR) proteins [4]. The adaptor proteins are apoptosis-associated speck-like proteins containing caspase activation and recruitment domain (ASC) proteins with a N-terminal pyrin domain (PYD) and a C-terminal caspase activation and recruitment domain (CARD). Finally, the effector proteins are proteolytic caspase-1/-11 (mice)/-4 (human)/-5 (human). Inflammasomes are initiated by a diverse array of stimuli which activate sensor receptors within the cells, leading to their oligomerization and formation of a protein complex with ASC proteins. ASC proteins bridge sensor proteins and effector proteins via homotypic PYD-PYD and CARD-CARD interactions to form a large filamentous scaffold [5, 6]. Inactive caspase monomers are recruited to the ASC filaments and become self-activated [7]. The sensor-ASC-caspase macromolecular complex can be visualized as a speck of 1-2 *μ*M within the cytosol, which is considered a characteristic of inflammasome assembly [8]. Activated caspases cleaved prointerleukin-(IL-) 1*β*, pro-IL-18, and gasdermin D (GSDMD), resulting in the pyroptosis of the cell [9]. Pyroptosis is a catastrophic form of cell death with morphological characteristics of apoptosis and necrosis. Cell lysis occurs due to GSDMD-dependent pore formation in the cell membrane, disruption of the cellular ionic gradient, water influx, and cell swelling. This further leads to intensive inflammasome activation; release of cell components including damaged DNA, mitochondria, and enzymes; and finally cellular disruption of adjacent cells [9, 10]. There are five receptors known to assemble inflammasomes, including the NLR protein members NLRP1, NLRP3, and NLRC4, as well as ALR protein members absent in melanoma 2 (AIM2) and pyrin. Other proteins, including NLRP2, NLRP6, NLRP7, NLRP12, NLRP9a, RIG-I (retinoic acid-inducible gene I), and IFI16 (interferon-*γ*-inducible protein 16), can also activate caspase-1, though the underlying mechanism which is less thoroughly explored [11–16].

### 1.1. NLRP3 Inflammasomes

The NLRP3 inflammasome is the most studied type, and genome-wide associated studies show that the mutation in the human *NLRP3* gene is linked to hereditary cryopyrin-associated periodic syndrome (CAPS), a spectrum of clinical manifestations including Muckle-Wells syndrome, familiar cold autoinflammatory syndrome, and neonatal-onset multisystem inflammatory disease [17]. It is also believed that NLRP3 inflammasomes are closely related to the onset of many diseases, including kidney diseases, cardiovascular diseases, rheumatoid arthritis (RA), asthma, gout, HIV infection, and Alzheimer's disease [18]. The NLRP3 protein consists of a C-terminal leucine-rich repeat (LRR) domain, a nucleotide-binding domain (NBD) in the middle, and a N-terminal PYD domain. The assembly of NLRP3 inflammasomes requires two signals. The first priming signal requires the engagement of toll-like receptors (TLRs), nucleotide-binding oligomerization domain (NOD) 2, or a tumor necrosis factor (TNF) receptor with specific ligands and cytokines. All of these signals activate NF-*κ*B and thus increase the expression of NLRP3, pro-caspase-1, pro-IL-1*β*, and pro-IL-18. The second signal required is that NLRP3 molecules sense a variety of danger signals and recruit other components to form the macromolecular complex [3]. These danger signals include pathogens such as *Staphylococcus aureus*, *Listeria monocytogenes*, *Escherichia coli*, *Sendai virus*, and *Influenza virus*, as well as DAMPs such as uric acid crystal, silica crystals, asbestos, alum, and X-ray. Previously, it seemed that potassium efflux was a downstream convergence point for the NLRP3 inflammasome assembly triggered by these diverse signals [19]. The molecular basis for the efflux K^+^ signal relies on a series of events including the activation of purinergic receptor P2X7 via ATP from dying cells, unstable mitochondria, integration of lysosomes, and the production of reactive oxygen species (ROS) [8]. However, it has been recently found that K^+^ signals are not necessary for the formation of NLRP3. NIMA-related kinase 7 can directly bind to the LRR domain of NLRP3 and controls the formation of the NLRP3 macromolecules [20, 21]. Recently, it was found that phosphatidylinosito-4-phosphate (PtdIns4P) recruited NLRP3 to the trans-Golgi network which served as a scaffold for NLRP3 aggregation, while disruption of the interaction between NLRP3 and PtdIns4P on the trans-Golgi network blocked NLRP3 aggregation and downstream signaling [22]. Also, cathepsins and cytoskeleton destabilization have been implicated in NLRP3 inflammasome activation [23, 24]. After its activation, NLRP3 proteins bind to ASC proteins via the PYD region, then ASC proteins in turn recruit pro-caspase-1 proteins with the same CARD region, cleaving it into mature caspase-1 composed of p10 and p20 subunits. Active caspase-1 then processes pro-IL-1*β* and pro-IL-18 into mature IL-1*β* and IL-18, and cuts GSDMD into N-terminal and C-terminal fragments. GSDMD-N of GSDMD binds to phosphoinositides and cardiolipin presented in the mammalian cell plasma, creating extensive membrane pores with an inner diameter of 12-14 nm, causing leakage of IL-1*β* and IL-18 and other cell components [9, 25, 26]. An alternative activation pathway for NLRP3 inflammasomes involves caspase-11/-4/-5 directly recognizing LPS, creating macromolecules and cutting GSDMD to release N-terminus, and thus forming membrane pores. Caspase-11 can also cleave the pannexin 1 channel protein, resulting in ATP leakage, activation of P2X7, influx of Ca^2+^, efflux of K^+^, and finally NLRP3 inflammasome activation and pyroptosis [9, 27, 28].

### 1.2. NLRP1 Inflammasomes

The NLRP1 inflammasome was first found responding to the *Bacillus anthracis* lethal factor. Humans only have one NLRP1 protein, whereas mice have NLRP1a, NLRP1b, and NLRP1c paralogues, all of which lack a PYD domain. The human NLRP1 protein contains NBD, LRR, a function to find domain (FIIND), and C-terminal CARD regions. Recently, it was found that functional degradation of NLRP1 led to inflammasome activation by diverse pathogen enzymes. First, autoprocessing of FIIND domain generates two associated fragments. Secondly, NLRP1b is cleaved by the lethal factor of *B. anthracis* or ubiquitinated by *Shigella* lpaH7.8, targeting for proteasome degradation. Finally, the release of bioactive C-terminal of NLRP1b triggers inflammasome assembly [29, 30]. At the other hand, experiments have shown that mouse NLRP1a protein has inflammasome functions since a point mutation at aa593 Q→P caused a strong systemic inflammatory response, driven by caspase-1 and IL-1*β*, while *Nlrp1a*-deficient mice showed enhanced recovery from hematopoietic and infectious stress [31–34]. Besides *B. anthracis*, NLRP1 has also been implicated in the host response to protozoan *Toxoplasma gondii*. Mice deficient in *Nlrp1b* and *Nlrp3* produced less IL-1*β* and IL-18 upon *T. gondii* infection, as they harbored increased parasitic loads [35].

### 1.3. NLRC4 Inflammasomes

The NLRC4 inflammasome contains NLRC4 and NLR apoptosis inhibitory proteins (NAIPs). NAIP family proteins directly bind to a bacterium's type 3 secretion system (T3SS) and flagellin, and then become activated, allowing them to recruit and activate NLRC4. Humans only have one NAIP protein, whereas mice have several members, including NAIP1, NAIP2, and NAIP6. The ligand specificity differs in mouse and human NLRC4 inflammasomes. Mouse NAIP isoforms recognize flagellin, T3SS rod, and needle proteins, while the human NAIP is activated by T3SS needle proteins [36]. The activation of NAIP proteins attracts and activates NLRC4, which in turn attracts caspase-1 either directly or indirectly through ASC, causing inflammatory responses [37–40].

### 1.4. AIM2 Inflammasomes

ALR proteins, which belong to the IFI family, have also been implicated in inflammasome activation and type I interferon response. Humans have 4 ALR genes, including AIM2, IFI16, pyrin-1, and myeloid cell nuclear differentiation antigen, whereas mice have 13 ALR genes [41]. AIM2 recognizes exogenous DNA of bacteria (e.g., *Listeria monocytogenes*) and viruses (e.g., *Papillomavirus*), as well as endogenous DNA within the cells, triggering inflammasome activation and interferon synthesis [42, 43]. ALR proteins contain HIN200 and PYD regions, for which the HIN200 region directly binds to DNA while the PYD region mediates protein-protein interaction [44, 45]. Mouse p202/human IFI16 proteins serve as negative regulators of AIM2 inflammasomes by inhibiting the formation of the AIM2-ASC complex [46–49].

### 1.5. Pyrin Inflammasomes

Pyrin is coded by the *MEFV* gene, and its mature form includes a PYD, two B-boxes, and a coiled-coil domain. The human pyrin protein also includes a B30.2 domain. Pyrin can sense signals from *Clostridium difficile* TcdB, *Clostridium botulinum* C3, and *Vibrio parahaemolyticus* VopS proteins. Pyrin does not directly interact with the aforementioned signals; instead, these signals cause various modifications (glycosylation, adenylylation, ADP-ribosylation, etc.) of Rho GTPases, causing the rearrangement of the cytoskeleton and subsequent activation of pyrin inflammasomes [50–52].

## 2. Research in Inflammasome-Related Kidney Diseases

Inflammatory responses exist in almost all kinds of kidney diseases, which are consequences of immune cells sensing PAMPs and DAMPs. It is believed that innate immune systems participate more actively than adaptive immune systems in recognizing and responding to PAMPs and DAMPs in kidney, maintaining homeostasis by regulating endogenous processes like inflammation and apoptosis [53]. PAMPs, such as virus and bacteria, are closely linked to pathogenesis of kidney diseases. For example, acute poststreptococcal glomerulonephritis is the leading cause of glomerulonephritis in children and is mainly caused by group A *Streptococcus* [54]. Hepatitis B virus triggers IgA nephropathy (IgAN) and membranous nephropathy, while measles and dengue fever virus are linked to acute interstitial nephritis [55]. DAMPs derived from endogenous molecules released from dying cells (e.g., amyloid-*β*, high-mobility group box 1 protein, ATP, uric acid crystals, chromatin, and DNA) also activate cellular receptors, leading to downstream inflammation resembling PAMPs-triggered signaling pathways [56].

The innate immune defense is characterized by PRR families of membranouse and cellular receptors (TLRs, RIG-I receptors, NOD receptors, scavenger receptors, C-type lectin, etc.) recognizing PAMPs and DAMPs, then initiating inflammation which primarily include interferon-(IFN-) *α* and IFN-*β*, as well as proinflammatory cytokines TNF-*α*, IL-1*β*, IL-6, IL-18, etc. [57]. Within this, innate immune cells (e.g., macrophages, monocytes, and dendritic cells) frequently employ inflammasomes to trigger the synthesis of IL-1*β* and IL-18 [1]. In the kidneys, IL-1*β* and IL-18 cause renal injury after binding to their receptors, which are abundantly expressed on infiltrated leukocytes, renal endothelial cells, and tubular epithelial cells [58–62]. IL-1*β* and IL-18 can activate MAPK and NF-*κ*B signaling pathways, thus leading to the production of nitric oxide, cyclooxygenase-2, inflammatory cytokines, and superoxide products [58, 63–65], finally exacerbating renal inflammation. Also, IL-1*β* and IL-18 promote the expression of adhesion molecules such as vascular cell adhesion molecule-1, intercellular cell adhesion molecule-1 [58, 66], and vascular endothelial growth factor [67], which cause systemic endothelial dysfunction (ED), a process that promotes leukocyte adhesion and vascular leakage in the kidney. Besides the IL-1*β*/IL-18 axis, other proinflammatory mediators (e.g., cytokines, ROS, bioactive lipids, and adhesion molecules) derived from inflammatory responses also contribute to ED, aberrant extracellular matrix metabolism, proliferation of renal resident cells, activation of coagulation system, and receptor-mediated vasoreactivity, finally leading to tubular injury, nephron dropout, and kidney function deterioration [68].

Besides immune cells, resident kidney cells also take part in inflammation and the tissue repair process. Intrarenal cells respond to circulating proinflammatory mediators by amplifying production of ROS and other proinflammatory factors [68]. Intrinsic renal cells (epithelial cells, podocytes, and endothelial cells) express components of the inflammasome pathway, synergically contributing to renal inflammation [61]. Evidence demonstrates positive expressions of *NLRP2*, *NLRP3*, *NLRP6*, *NLRP10*, and *NLRP12* in human kidney samples [53]. The expression of *NLRP3* was confirmed in both tubular cells and podocytes, and it was increased in biopsies of human kidney diseases (hypertension kidney injury, acute tubular necrosis, diabetic nephropathy, IgAN, and lupus nephritis) [69]. Most of inflammasome research is focused on NLRP3 inflammasomes, though the roles of other inflammasomes are also important in the pathogenesis of inflammatory kidney diseases. Below, we review recent research on inflammasomes relating to various kidney diseases (Table 1), to understand the role inflammasomes play in them and to hope to provide clues to new therapeutic strategies.

### 2.1. Inflammasomes in Acute Kidney Injury

Acute kidney injury (AKI) is acute, but reversible kidney function deterioration in a short period caused by ischemia, sepsis, and renal toxins. Research suggests that AKI accompanies large amounts of cell apoptosis and necrosis, as well as the release of cell debris. The heat shock proteins, histones, and high-mobility group B1 proteins in the cell debris can activate NF-*κ*B via TLR2 and TLR4 in a MyD88-dependent pathway to promote the expression of NLRP3 and pro-IL-1*β*, therefore activating the inflammasome pathway [70–72]. Hydroxychloroquine (TLR7/8/9 inhibitors) blocked the priming and activation of NLRP3 by downregulating NF-*κ*B signaling and activity of cathepsins B and L, thus attenuating renal dysfunction in an ischemia-reperfusion (I-R) model [73]. In the murine acute kidney injury model, a lack of NLRP3 resulted in protected kidney functions, attenuated inflammation, and increased survivability of mice [74]. In the murine I-R injury model, Shigeoka et al. found that *Nlrp3*-/- mice demonstrated decreased mature forms of IL-1*β*, IL-18, and caspase-1, alleviated renal function damage, tubular necrosis, and leukocyte infiltration [75]. However, no difference was found when comparing *Il-18*-/- mice, *Il-1r*-/-mice, *caspase1*-/- mice, and *Asc*-/- mice to regular mice after I-R injury, which indicated a new functional role of NLRP3. They also found that in the I-R injury model, *Nlrp3*-/- mice had much less apoptosis than wild-type and *Asc-/-* mice [75]. All evidence agrees with the proinflammatory role of the NLRP3 molecule in AKI, though the interactions of apoptosis, inflammasomes, and pyroptosis are intertwined. For instance, the apoptotic caspase-8 and its adaptor are required for priming and activation of NLRP3 inflammasomes [76]. In human monocytes, LPS signaling can recruit caspase-8 to trigger NLRP3 activation in a K^+^ efflux-independent manner while the apoptosis signal can induce a K^+^ efflux-dependent inflammasome activation [77]. Recently, two groups have found that *Yersinia* infection recruited caspase-8 to cleave GSDMD at Asp276, leading to cell pyroptosis but not apoptosis [78, 79]. This event happened before the activation of NLRP3 inflammasomes and the release of IL-1*β*. In brief, apoptosis limits cells from further cytokine production and tissue injury, while inflammasome-induced pyroptosis destroys injured cells but releases proinflammatory cytokines. The regulation and switching of these events are fine-tuned and controlled under different pathological situations [36].

### 2.2. Inflammasomes in Chronic Kidney Disease

Chronic kidney disease (CKD) develops over several years without obvious clinical symptoms, but results in irreversible kidney damage, ultimately leading to end-stage renal disease. CKD is characterized by leukocyte infiltration, proinflammatory cytokine secretion, cell damage/death, fibrosis, and renal function failure as a common end. Persistent, low-grade chronic inflammation has been recognized as an essential part of CKD. Biomarkers of inflammation were inversely correlated with the estimated glomerular filtration rate (eGFR) in CKD patients [80]. The unilateral ureteral obstructive (UUO) model is commonly used to study renal fibrosis in CKD. In this model, *Nlrp3*-deficient mice demonstrated less damage regarding of tubular injury, inflammation, and fibrosis, as well as less activation of caspase-1 and release of mature IL-1*β* and IL-18. Furthermore, it has been proven that myeloid cells and nonmyeloid cells both play important roles in NLRP3-mediated renal fibrosis by chimeric mouse experiments [36]. Additionally, *Aim2-/-* mice exhibited attenuated renal injury, fibrosis, and inflammation compared with wild-type littermates in the UUO model. In terms of mechanisms, researchers found that DNA released from necrotic cells drives the activation of AIM2 inflammasomes in macrophages, thus promoting tissue injury in the kidney [81].

### 2.3. Inflammasomes in IgA Nephropathy

NLRP3 expression in the kidney was increased in patients of IgAN [82]. In normal kidneys, NLRP3 expression was detected in kidney tubular epithelial cells, while it was found with increased amounts in renal tissue and glomeruli of IgAN. However, increased NLRP3 expression was associated with better clinical outcome in IgAN, for reasons still unclear [69, 82]. In an accelerated and progressive IgAN model, compound antroquinonol and osthole can suppress ROS production and NLRP3 activation, thus reducing mesangial cell proliferation, glomerular sclerosis, and lymphocyte infiltration [83, 84]. In the same murine model, *Nlrp3*-deficient mice demonstrated less leukocyte infiltration, better renal functions, and less caspase-1, IL-1*β*, and IL-18 synthesis; while their Th17 ratios were decreased, and Treg ratios were increased [85]. The mechanism was explained as follows: IgA-IC can induce the activation of NLRP3 inflammasomes in macrophages, dendritic cells, and renal mesangial cells, causing the release of IL-1*β* and IL-18. Hence, suppressing the activation of NLRP3 decreased inflammatory response [85]. These results demonstrated that activation of NLRP3 inflammasomes in both innate immune cells and kidney-resident cells led to kidney damage in IgAN.

The expression profiles of inflammasome components in the peripheral blood of IgAN patients were lacking. Here, we analyze the mRNA levels of eleven members of the inflammasome pathway (*NLRP3*, *NLRP1*, *NLRC4*, *NAIP*, *AIM2*, *IFI16*, *PYRIN*, *ASC*, *caspase-1*, *IL-1β*, and *IL-18*) from peripheral blood mononuclear cells (PBMCs) in IgAN patients, and we found that mRNA levels of *NLRP3* were significantly increased in PBMCs of IgAN patients (Table 2). Considering previous data showing that *NLRP3* expression was increased in the kidneys of IgAN patients, increased expression of *NLRP3* in PBMCs strengthens the notion that NLRP3 is relevant to the pathogenesis of IgAN, both in renal resident cells and in myeloid cells.

### 2.4. Inflammasomes in Diabetic Nephropathy

In diabetic nephropathy (DN), NLRP3 inflammasomes promoted disease onset and progress under high-glucose conditions [86]; meanwhile, IL-1*β* and IL-18 secreted from both immune cells and glomerular resident cells exaggerated disease severity [87–89]. Indeed, the lack of NLRP3 or blockade of IL-1R mitigated the symptoms of diabetic mice [89]. It was found that the immunostaining of P2X4, NLRP3, IL-1*β*, and IL-18 was sharply increased in renal tubular epithelial cells from patients of DN [90]. Moreover, the ATP-P2X4 and TLR4 signaling pathway closely controlled the expression, as well as the activation of NLRP3 inflammasomes [90, 91]. In addition to tubular epithelial cells, NLRP3 was also detected in murine cultured podocytes and human kidneys with mild DN [92]. Considering recent evidence demonstrating their positive MHCII expression and antigen-presenting capacity, podocytes may have similar functions as renal dendritic cells and kidney-infiltrating macrophages, contributing to the pathogenesis of DN and other inflammatory renal diseases [93, 94]. When exposed to high-glucose environments, podocytes significantly produced ROS, which is key to the activation of NLRP3 inflammasomes [89]. Meanwhile, another pro-oxidative factor thioredoxin-interacting protein (TXNIP) activated NLRP3 inflammasomes by interacting with NLRP3 in high glucose-treated podocytes [95]. In addition, LPS worked synergically with high glucose to induce the production of ROS and IL-1*β* in renal cells, indicating that ROS/TXNIP/NLRP3/IL-1*β* pathways are highly relevant in the development of DN [96, 97]. Recently, it was found that *Nlrc4* deficiency also resulted in diminished renal injury in a murine diabetic model [98]. NLRC4 inflammasomes augmented NF-*κ*B activation, IL-1*β* release, and macrophage infiltration in diabetic mice, in parallel to NLRP3 inflammasomes [98].

### 2.5. Inflammasomes in Lupus Nephritis

**Figure 1:**
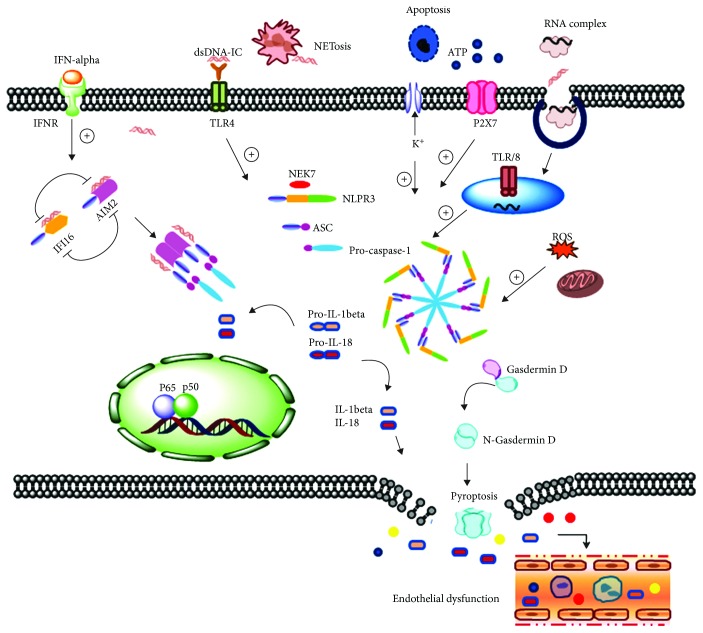
Schematic illustration of the role of inflammasomes in lupus nephritis. ATP released from dead cells activates P2X7 on the cell membrane, inducing the K^+^ efflux and NLRP3 inflammasome assembly. The assembly of NLRP3 inflammasomes and maturation of caspase-1 caused the cleavage of pro-IL-18 and pro-IL-1*β*, as well as the release of the N-terminal gasdermin D to induce pyroptosis. The neutrophil extracellular trap releases a large amount of dsDNA and other cellular components and induces the formation of a dsDNA-containing immune complex, which promotes the activation of NLRP3 inflammasomes via TLRs. The endogenous RNA-containing complex can also activate NLRP3 inflammasomes through the endosome-located TLR7/8 signaling pathway. AIM2 and another IFI family member IFI16 can sense/recognize dsDNA, leading to the activation of caspase-1 and maturation of IL-18 and IL-1*β*. The signature cytokine of lupus, IFN-*α*, binds to IFN receptors on the cell surface, which regulate the expression and maximum activity of AIM2 inflammasomes. Released IL-1*β*, IL-18, and other inflammatory mediators resulted in endothelial leakage, immune cell infiltration, and finally tissue inflammation and functional deterioration of the kidney.

As an autoimmune disease, systemic lupus erythematosus is composed of a series of immune aberrances, including abnormal T cell development, innate immune dysregulation, and increased B cell activity [93]. These events contribute to the occurrence of circulating double-stranded DNA- (dsDNA-) containing immune complexes and other nuclear component debris, as well as the production of the central cytokine mediator of lupus, IFN-*α* [99]. Lupus nephritis (LN), a major cause of morbidity of lupus, is induced by inflammation following deposition of the immune complex in the kidneys [100]. The roles of inflammasomes in lupus are complicated, and numerous molecules contribute to the pathogenesis of lupus as illustrated in Figure 1. Nuclear dsDNA derived from cell apoptosis, necrosis, and neutrophil extracellular trap leads to the formation of anti-dsDNA autoantibody [101–103]. These immune complexes can bind to TLRs and other cytosolic receptors, causing activation of NLRP3 inflammasomes, and in turn activation of caspase-1 and release of IL-1*β* and IL-18 [104, 105]. At the same time, ATP released from dead cells further hastens this process through P2X7 [105, 106]. New Zealand Black/New Zealand White hybrid F1 mice is a common model for a murine lupus study. In this model, T cells are poorly developed and inclined to apoptosis, and B cells produce high titers of anti-DNA antibodies and anti-nuclear antibodies, which causing mice often dying from severe glomerular nephritis [107, 108]. NZM2328 mice, derived from NZB mice, also developed self-reactive antibodies and glomerular nephritis [109]. According to research on this model, it has been found that blockade of NLRP3 inflammasomes resulted in abated LN symptoms, impaired IL-1*β* release, and improved kidney functions [104]. At the same time, it was found that the P2X7 inhibitor decreased the protein expression of NLRP3 and ASC, therefore reducing IL-1*β* release, anti-dsDNA antibody concentration, and symptoms of LN [110, 111]. Moreover, this model revealed that NLRP3 inflammasomes were activated in podocytes via ROS production, while similar evidence was found in the kidney biopsies of patients with LN [112]. In another LN model based on NZB mice combined with LPS injection, inhibiting ROS and NLRP3 inflammasome pathways also protected kidney functions, by alleviating cell apoptosis and renal histopathology [112]. In a murine lupus model induced by lupus serum, *Il-1r*-deficient mice and *caspase-1*-deficient mice demonstrated major improvements in skin inflammation, with decreased expression of MCP-1 and TNF-*α* [113], indicating inflammasome pathways contribute to skin inflammation of LN. All these results show that inflammasome-related molecules play roles in lupus progression, including LN. However, in the common *lpr* lupus model, the lack of NLRP3 and ASC did not deliver an expected effect on disease improvement, instead further damaging kidney function and causing exacerbated activation of lymphocytes [114]. Further research had shown that NLRP3 drove the expression of the TGF-*β* receptor and downstream molecules which can suppress lupus progression [114]. Another explanation is that the *lpr* lupus model is based on extensive cell apoptosis, and as mentioned before, the apoptosis signaling pathway interacted with the NLRP3 signaling pathway.

On the other hand, complement components were also found capable of influencing the activation of inflammasomes in lupus. Genomics research found that polymorphism of C1q was closely related to the pathogenesis of lupus and the lack of C1q promoted the development of lupus-like autoimmune diseases [115]. Evidence showed that C1q suppressed the NLRP3 inflammasome pathway, whereas it promoted the synthesis of anti-inflammatory cytokines IL-10 and IL-37 [116]. Meanwhile, it was reported that *NLRP1/IL-1β* polymorphism was correlated with the pathogenesis of autoimmune diseases including lupus [117–119]; however, exact evidence about how NLRP1 was involved in lupus was not illustrated.

The importance of dsDNA-sensing inflammasomes (AIM2, IFI16) in LN is also worth mentioning. AIM2 expression was first found to be related with colorectal cancer and prostate cancer [120–122]. Additionally, AIM2 expression was increased in autoimmune diseases, and dsDNA was recognized by AIM2 in keratinocytes to boost autoimmunity [123]. In lupus, increased *AIM2* expression was positively correlated with the disease's SLEDAI score and was regulated by body hormones [100, 124, 125]. Male hormones can increase the expression of *AIM2* in cells [125], and consistently, a higher level of *AIM2* mRNA in macrophages was observed in male patients with lupus compared with female patients [126]. In the LN model induced by apoptotic DNA, *AIM2* expression in macrophages showed a substantial increase, demonstrating a positive correlation to anti-dsDNA antibody titer. Injecting siAIM2 can reduce the activation of macrophages, thus diminishing renal inflammatory responses [100]. However, there is also contradictory evidence showing that AIM2 was negatively associated with inflammation in lupus. *Aim2* knockdown augmented type I IFN response induced by cytosolic DNA in macrophages [127]. Inhibition of AIM2 promoted the expression of another IFI member IFI16/p202 [128], which was found increased in leukocytes of lesion skin and peripheral blood from lupus patients [129–131]. IFI16/p202 conversely suppressed the activation of AIM2 inflammasomes by binding to the AIM2-ASC complex [46, 49, 128]. Moreover, the critical cytokine of lupus, IFN-*α*, can influence expression/activity of both AIM2 and IFI16 [132, 133]. Considering the antagonizing relationship of IFI16 and AIM2, how AIM2 and IFI16 work in lupus is worthy of further investigation.

## 3. Closing Remarks

From all of the above, it is clear that inflammasomes play key roles in inflammatory kidney diseases. At present, the most current research is still focused on NLRP3 inflammasomes. Glomerular dysfunction associated with inflammatory microenvironments may benefit from inhibiting NLRP3 inflammasomes, and many compounds have shown this effect in murine models of kidney diseases (Table 3). High-throughput screening and an *in vitro* engineered cell line have also been used for hunting specific compounds for inflammasomes [134, 135]. Convincing evidence from clinical trials also demonstrated that by blocking the inflammasome pathway, cellular inflammation and tissue damage are reduced. In human, antagonism of IL-1 signaling has been proved effective in several types of inflammatory diseases. Recombinant human IL-1ra (anakinra), inhibiting IL-1 binding to IL-1 receptors, has been successfully used in RA [136]. A monoclonal antibody against IL-1*β* (canakinumab) is beneficial in the treatment of RA and CAPS [137, 138]. An anti-IL-1*β* antibody (gevokizumab) has been tested in patients with DN since 2015 [139]. Also, antagonists of P2X7 have shown positive outcome in clinical trials of Crohn's disease [140] and RA [141, 142].

Much of our knowledge about inflammasomes is limited to experimental animal models, and the role of inflammasomes in kidney diseases still requires more intensive research. For instance, the effects of AIM2 and IFI16 in kidney diseases are very intriguing, though there is still no direct evidence proving how they play roles in the pathogenesis of nephritis. As many signals may influence the activation of inflammasomes, any molecule involved in the inflammasome pathway could be the key to therapeutic intervention of kidney diseases. Analyzing how these signals influence inflammasomes will provide much-needed evidence in understanding and curing kidney diseases.

## Figures and Tables

**Table 1:** Roles of inflammasomes in inflammatory kidney diseases.

Disease	Inflammasomes involved	Roles and potential mechanism	Reference
Acute kidney injury	NLRP3	*Nlrp3* gene deletion protected mice from AKI.	[143, 144]
		ATP-sensitive P2X7 receptor activates the NLRP3 inflammasomes.	[145]
		Cell debris (histones, HGBM1, etc.) mediated NLRP3 inflammasome activation.	[70, 72, 74]

IgA nephropathy	NLRP3	*Nlrp3* deficiency improved renal function and renal injury in a mouse IgAN model.	[85]
		*NLRP3* gene expression was correlated with clinical outcome in IgAN patients.	[82]
		IgA-immune complexes activated NLRP3 inflammasomes involving ROS production in macrophages, dendritic cells, and renal intrinsic cells.	[85]
		Generation of ROS and activation of NF-*κ*B lead to NLRP3 activation, which is a key event in IgAN.	[84]

Diabetic nephropathy	NLRP3	*Nlrp3*-deficient mice are protected against diabetic nephropathy.	[88, 89]
		Mitochondrial ROS, TLR4 signaling, and NLRP3 inflammasome activation aggravate diabetic nephropathy.	[89, 91]
		TXNIP activated NLRP3 inflammasomes in podocytes of diabetic nephropathy.	[95, 146]
		High glucose and LPS activate ROS/TXNIP/NLRP3/IL-1*β* inflammasome signaling in glomerular mesangial cells.	[96]
		ATP-P2X4 signaling mediated high glucose-induced activation of NLRP3 inflammasomes.	[90]
	NLRC4	*Nlrc4* deficiency resulted in diminished disease progression in diabetic mice. Activation of NF-*κ*B and MAPK pathways was blocked by *Nlrc4* deficiency.	[98]

Lupus nephritis	NLRP1	Polymorphism of *NLRP1* was related to the pathogenesis of lupus.	[119]
	NLRP3	NLRP3 inflammasomes were activated in podocytes from NZM2328 mice and patients of LN; P2X7/NLRP3 is a key signaling pathway.	[110, 111]
		Immune complex containing dsDNA induced IL-1*β* production through NLRP3 inflammasomes.	[104, 105]
		Lack of NLRP3 enhanced lupus symptom in B6^lpr^ mice by inhibiting TGF target genes.	[114]
	AIM2	*AIM2* expression was increased in lupus patients and closely correlated with the severity of disease in SLE patients. AIM2 facilitates the apoptotic DNA-induced lupus damage via arbitrating macrophage functional maturation.	[100, 131]
	IFI16	IFI16 expression was increased in leukocytes but not in kidney biopsies of lupus patients.	[129, 131]
		Anti-IFI16 antibody titers were higher in lupus patients and inversely correlated with proteinuria.	[110]

**Table 2:** Expression profiles of inflammasome members in PBMCs of IgAN patients.

	Healthy donors *n* = 17	IgAN patients *n* = 22	*P* value
Gender	F8/M9	F15/M7	0.19
Age	34.9 ± 9.3	32 ± 10.3	0.38
CREA	56.2 ± 10.4	87 ± 38.3	0.007^∗∗^
*NLRP1* mRNA levels	0.062 ± 0.003	0.068 ± 0.005	0.42
*NLRP3* mRNA levels	0.018 ± 0.0007	0.028 ± 0.003	0.014^∗^
*NLRC4* mRNA levels	0.016 ± 0.0010	0.015 ± 0.0015	0.64
*NAIP* mRNA levels	0.069 ± 0.006	0.07 ± 0.005	0.69
*AIM2* mRNA levels	0.0035 ± 0.0003	0.0032 ± 0.0005	0.68
*PYRIN* mRNA levels	0.07 ± 0.005	0.08 ± 0.009	0.30
*IFI16* mRNA levels	0.13 ± 0.012	0.10 ± 0.011	0.08
*ASC* mRNA levels	0.18 ± 0.02	0.16 ± 0.019	0.47
*Caspase-1* mRNA levels	0.17 ± 0.02	0.12 ± 0.002	0.41
*IL-1β* mRNA levels	0.015 ± 0.0016	0.011 ± 0.0012	0.10
*IL-18* mRNA levels	0.012 ± 0.002	0.010 ± 0.001	0.49

Verified healthy donors and primary IgAN patients were enrolled under the supervision of the Ethics Review Committee of the First Affiliated Hospital, Sun Yat-sen University (Guangzhou, China), and this study was conducted in accordance with the guidelines proposed in the Declaration of Helsinki. None of the patients had been treated with steroids and/or immunosuppressive drugs within one year nor did they show clinical infection symptoms at the day when blood samples were taken. PBMCs from venous blood with anticoagulant EDTA-K2 were enriched and subjected to RNA extraction. Gene expression was analyzed with real-time PCR analysis and calculated with the 2^-*ΔΔ*Ct^ method, using *GAPDH* as the internal control. Sequences and primers for each genes were acquired from the NCBI database (https://www.ncbi.nlm.nih.gov). All statistical assessments were two-sided using a significance value of *P* < 0.05 (indicated as ∗) and *P* < 0.01 (indicated as ∗∗).

**Table 3:** Compounds targeting inflammasomes for kidney diseases.

Compound	Inflammasome target	Disease/animal model	Reference
BAY 11-7082 (NF-*κ*B inhibitor)	NLRP3	Paraquat-induced acute kidney injury model (rat)	[147]
Allopurinol (oxidase inhibitor)	Need to be specified	Glycerol-induced acute kidney injury model (rat)	[148]
4-Hydroxycinnamaldehyde-galactosamine	NLRP3	LPS-induced renal inflammation (mice)	[149]
Harmine	NLRP3	LPS-induced renal inflammation model (mice)	[150]
Artemisinin	NLRP3	5/6 nephrectomy (rat)	[151]
Rotenone (inhibitor of mitochondrial complex I)	NLRP3	Aldosterone-infused renal nephropathy model (rat)	[152]
Hydroxychloroquine	NLRP3	Ischemia-reperfusion model (mice)	[73]
1,3-Butanediol (inhibitor of the NLRP3)	NLRP3	Nephrocalcinosis-related chronic kidney disease model (mice)	[153]
CP-456773 (inhibitor of the NLRP3)	NLRP3	Oxalate- or adenine-induced crystal nephropathy	[154]
Ginsenoside compound K	NLRP3	High-fat diet/streptozotocin-induced diabetic nephritis (mice)	[155]
		Unilateral ureteral obstruction model (mice)	[156]
FL-926-16 (carnosine derivative)	NLRP3	db/db diabetic mice	[157]
Verapamil	NLRP3	Diabetic nephropathy	[158]
Osthole	NLRP3	A progressive IgAN model (mice)	[84]
Antroquinonol			[83]
Resveratrol			[159]
Citral	NLRP3	LPS-induced accelerated and severe lupus nephritis model (mice)	[112]
Piperine	NLRP3	Pristine-induced lupus nephritis (mice)	[160]
Curcumin	NLRP3	Lupus-prone female MRL/lpr mice	[161]
Brilliant blue G (P2X7 antagonist)	NLRP3	NZM2328 lupus-prone mice	[110]
MCC950 (inhibitor of NLRP3)	NLRP3	NZM2328 lupus-prone mice	[111]

## Abbreviations

AIM2: Absent in melanoma 2
AKI: Acute kidney injury
ALR: AIM2-like receptor
ASC: Apoptosis-associated speck-like protein containing a CARD
CAPS: Cryopyrin-associated periodic syndrome
CARD: Caspase activation and recruitment domain
CKD: Chronic kidney disease
DAMPs: Damage-associated molecular patterns
DN: Diabetic nephropathy
dsDNA: Double-stranded DNA
ED: Endothelial dysfunction
eGFR: Estimated glomerular filtration rate
FIIND: Function to find domain
GADMD: Gasdermin D
IgAN: IgA nephropathy
NAIP: NLR apoptosis inhibitory protein
NBD: Nucleotide-binding domain
NLR: NOD-like receptor
NLRP: Nucleotide-binding oligomerization domain, leucine-rich repeat, and pyrin domain containing protein
NOD: Nucleotide-binding oligomerization domain
IFN: Interferon
IL: Interleukin
I-R: Ischemia-reperfusion
IFI: Interferon-*γ*-inducible protein
LRR: Leucine-rich repeats
LN: Lupus nephritis
PAMPs: Pathogen-associated molecular patterns
PBMCs: Peripheral blood mononuclear cells
PRRs: Pattern recognition receptors
PYD: Pyrin domain
TLR: Toll-like receptors
TNF: Tumor necrosis factor
TXNIP: Thioredoxin-interacting protein
T3SS: Type 3 secretion system
RA: Rheumatoid arthritis
RIG-I: Retinoic acid-inducible gene I
ROS: Reactive oxygen species
UUO: Unilateral ureteral obstruction.

## References

[1] Martinon F., Burns K., Tschopp J. (2002). The inflammasome: a molecular platform triggering activation of inflammatory caspases and processing of proIL-*β*. *Molecular Cell*.

[2] Santana P. T., Martel J., Lai H. C. (2016). Is the inflammasome relevant for epithelial cell function?. *Microbes and Infection*.

[3] Mathur A., Hayward J. A., Man S. M. (2018). Molecular mechanisms of inflammasome signaling. *Journal of Leukocyte Biology*.

[4] Mariathasan S., Monack D. M. (2007). Inflammasome adaptors and sensors: intracellular regulators of infection and inflammation. *Nature Reviews Immunology*.

[5] De Zoete M. R., Palm N. W., Zhu S., Flavell R. A. (2014). Inflammasomes. *Cold Spring Harbor Perspectives in Biology*.

[6] Broz P., Dixit V. M. (2016). Inflammasomes: mechanism of assembly, regulation and signalling. *Nature Reviews Immunology*.

[7] Lu A., Magupalli V. G., Ruan J. (2014). Unified polymerization mechanism for the assembly of ASC-dependent inflammasomes. *Cell*.

[8] Kesavardhana S., Kanneganti T. D. (2017). Mechanisms governing inflammasome activation, assembly and pyroptosis induction. *International Immunology*.

[9] Shi J., Gao W., Shao F. (2017). Pyroptosis: gasdermin-mediated programmed necrotic cell death. *Trends in Biochemical Sciences*.

[10] Miao E. A., Rajan J. V., Aderem A. (2011). Caspase-1-induced pyroptotic cell death. *Immunological Reviews*.

[11] Rathinam V. A. K., Fitzgerald K. A. (2016). Inflammasome Complexes: emerging mechanisms and effector functions. *Cell*.

[12] Elinav E., Strowig T., Kau A. L. (2011). NLRP6 inflammasome regulates colonic microbial ecology and risk for colitis. *Cell*.

[13] Kerur N., Veettil M. V., Sharma-Walia N. (2011). IFI16 acts as a nuclear pathogen sensor to induce the inflammasome in response to Kaposi sarcoma-associated herpesvirus infection. *Cell Host & Microbe*.

[14] Minkiewicz J., De Rivero Vaccari J. P., Keane R. W. (2013). Human astrocytes express a novel NLRP2 inflammasome. *Glia*.

[15] Vladimer G. I., Weng D., Paquette S. W. M. (2012). The NLRP12 inflammasome recognizes *Yersinia pestis*. *Immunity*.

[16] Poeck H., Bscheider M., Gross O. (2010). Recognition of RNA virus by RIG-I results in activation of CARD9 and inflammasome signaling for interleukin 1*β* production. *Nature Immunology*.

[17] Kuemmerle-Deschner J. B., Lohse P., Koetter I. (2011). *NLRP3* E311K mutation in a large family with Muckle-Wells syndrome - description of a heterogeneous phenotype and response to treatment. *Arthritis Research & Therapy*.

[18] Iwata M., Ota K. T., Duman R. S. (2013). The inflammasome: pathways linking psychological stress, depression, and systemic illnesses. *Brain, Behavior, and Immunity*.

[19] Groß C. J., Mishra R., Schneider K. S. (2016). K^+^ efflux-independent NLRP3 inflammasome activation by small molecules targeting mitochondria. *Immunity*.

[20] He Y., Zeng M. Y., Yang D., Motro B., Núñez G. (2016). NEK7 is an essential mediator of NLRP3 activation downstream of potassium efflux. *Nature*.

[21] Shi H., Wang Y., Li X. (2016). NLRP3 activation and mitosis are mutually exclusive events coordinated by NEK7, a new inflammasome component. *Nature Immunology*.

[22] Chen J., Chen Z. J. (2018). PtdIns4P on dispersed *trans*-Golgi network mediates NLRP3 inflammasome activation. *Nature*.

[23] Campden R. I., Zhang Y. (2019). The role of lysosomal cysteine cathepsins in NLRP3 inflammasome activation. *Archives of Biochemistry and Biophysics*.

[24] Joshi H., Morley S. C. (2019). Cells under stress: the mechanical environment shapes inflammasome responses to danger signals. *Journal of Leukocyte Biology*.

[25] Ding J., Wang K., Liu W. (2016). Pore-forming activity and structural autoinhibition of the gasdermin family. *Nature*.

[26] Shi J., Zhao Y., Wang K. (2015). Cleavage of GSDMD by inflammatory caspases determines pyroptotic cell death. *Nature*.

[27] Yang D., He Y., Munoz-Planillo R., Liu Q., Nunez G. (2015). Caspase-11 requires the pannexin-1 channel and the purinergic P2X7 pore to mediate pyroptosis and endotoxic shock. *Immunity*.

[28] Broz P., Monack D. M. (2013). Noncanonical inflammasomes: caspase-11 activation and effector mechanisms. *PLoS Pathogens*.

[29] Mitchell P. S., Sandstrom A., Vance R. E. (2019). The NLRP1 inflammasome: new mechanistic insights and unresolved mysteries. *Current Opinion in Immunology*.

[30] Sandstrom A., Mitchell P. S., Goers L., Mu E. W., Lesser C. F., Vance R. E. (2019). Functional degradation: a mechanism of NLRP1 inflammasome activation by diverse pathogen enzymes. *Science*.

[31] Masters S. L., Gerlic M., Metcalf D. (2012). NLRP1 inflammasome activation induces pyroptosis of hematopoietic progenitor cells. *Immunity*.

[32] Levinsohn J. L., Newman Z. L., Hellmich K. A. (2012). Anthrax lethal factor cleavage of Nlrp1 is required for activation of the inflammasome. *PLoS Pathogens*.

[33] Ewald S. E., Chavarria-Smith J., Boothroyd J. C. (2014). NLRP1 is an inflammasome sensor for *Toxoplasma gondii*. *Infection and Immunity*.

[34] Chavarría-Smith J., Vance R. E. (2013). Direct proteolytic cleavage of NLRP1B is necessary and sufficient for inflammasome activation by anthrax lethal factor. *PLoS Pathogens*.

[35] Zamboni D. S., Lima-Junior D. S. (2015). Inflammasomes in host response to protozoan parasites. *Immunological Reviews*.

[36] Krakauer T. (2019). Inflammasomes, autophagy, and cell death: the trinity of innate host defense against intracellular bacteria. *Mediators of Inflammation*.

[37] Zhao Y., Yang J., Shi J. (2011). The NLRC4 inflammasome receptors for bacterial flagellin and type III secretion apparatus. *Nature*.

[38] Zhao Y., Shao F. (2015). The NAIP–NLRC4 inflammasome in innate immune detection of bacterial flagellin and type III secretion apparatus. *Immunological Reviews*.

[39] Tenthorey J. L., Kofoed E. M., Daugherty M. D., Malik H. S., Vance R. E. (2014). Molecular basis for specific recognition of bacterial ligands by NAIP/NLRC4 inflammasomes. *Molecular Cell*.

[40] Miao E. A., Mao D. P., Yudkovsky N. (2010). Innate immune detection of the type III secretion apparatus through the NLRC4 inflammasome. *Proceedings of the National Academy of Sciences of the United States of America*.

[41] Brunette R. L., Young J. M., Whitley D. G., Brodsky I. E., Malik H. S., Stetson D. B. (2012). Extensive evolutionary and functional diversity among mammalian AIM2-like receptors. *Journal of Experimental Medicine*.

[42] Gray E. E., Winship D., Snyder J. M., Child S. J., Geballe A. P., Stetson D. B. (2016). The AIM2-like receptors are dispensable for the interferon response to intracellular DNA. *Immunity*.

[43] Jin T., Perry A., Jiang J. (2012). Structures of the HIN domain:DNA complexes reveal ligand binding and activation mechanisms of the AIM2 inflammasome and IFI16 receptor. *Immunity*.

[44] Hornung V., Ablasser A., Charrel-Dennis M. (2009). AIM2 recognizes cytosolic dsDNA and forms a caspase-1-activating inflammasome with ASC. *Nature*.

[45] Fernandes-Alnemri T., Yu J. W., Datta P., Wu J., Alnemri E. S. (2009). AIM2 activates the inflammasome and cell death in response to cytoplasmic DNA. *Nature*.

[46] Wang P. H., Ye Z. W., Deng J. J. (2018). Inhibition of AIM2 inflammasome activation by a novel transcript isoform of IFI16. *EMBO Reports*.

[47] Ru H., Ni X., Zhao L. (2013). Structural basis for termination of AIM2-mediated signaling by p202. *Cell Research*.

[48] Yin Q., Sester D. P., Tian Y. (2013). Molecular mechanism for p202-mediated specific inhibition of AIM2 inflammasome activation. *Cell Reports*.

[49] Veeranki S., Duan X., Panchanathan R., Liu H., Choubey D. (2011). IFI16 protein mediates the anti-inflammatory actions of the type-I interferons through suppression of activation of caspase-1 by inflammasomes. *PLoS One*.

[50] Chae J. J., Wood G., Masters S. L. (2006). The B30.2 domain of pyrin, the familial Mediterranean fever protein, interacts directly with caspase-1 to modulate IL-1*β* production. *Proceedings of the National Academy of Sciences of the United States of America*.

[51] Xu H., Yang J., Gao W. (2014). Innate immune sensing of bacterial modifications of Rho GTPases by the pyrin inflammasome. *Nature*.

[52] Kim M. L., Chae J. J., Park Y. H. (2015). Aberrant actin depolymerization triggers the pyrin inflammasome and autoinflammatory disease that is dependent on IL-18, not IL-1*β*. *Journal of Experimental Medicine*.

[53] Anders H. J., Muruve D. A. (2011). The inflammasomes in kidney disease. *Journal of the American Society of Nephrology*.

[54] VanDeVoorde R. G. (2015). Acute poststreptococcal glomerulonephritis: the most common acute glomerulonephritis. *Pediatrics in Review*.

[55] Anders H. J., Lichtnekert J., Allam R. (2010). Interferon-*α* and -*β* in kidney inflammation. *Kidney International*.

[56] Rosin D. L., Okusa M. D. (2011). Dangers within: DAMP responses to damage and cell death in kidney disease. *Journal of the American Society of Nephrology*.

[57] Wu J., Chen Z. J. (2014). Innate immune sensing and signaling of cytosolic nucleic acids. *Annual Review of Immunology*.

[58] Gerdes N., Sukhova G. K., Libby P., Reynolds R. S., Young J. L., Schonbeck U. (2002). Expression of Interleukin (IL)-18 and functional il-18 receptor on human vascular endothelial cells, smooth muscle cells, and macrophages. *Journal of Experimental Medicine*.

[59] Miyauchi K., Takiyama Y., Honjyo J., Tateno M., Haneda M. (2009). Upregulated IL-18 expression in type 2 diabetic subjects with nephropathy: TGF-*β*_1_ enhanced IL-18 expression in human renal proximal tubular epithelial cells. *Diabetes Research and Clinical Practice*.

[60] Nakamura S., Otani T., Okura R. (2000). Expression and responsiveness of human interleukin-18 receptor (IL-18R) on hematopoietic cell lines. *Leukemia*.

[61] Anders H. J. (2016). Of inflammasomes and alarmins: IL-1*β* and IL-1*α* in kidney disease. *Journal of the American Society of Nephrology*.

[62] Garlanda C., Dinarello C. A., Mantovani A. (2013). The interleukin-1 family: back to the future. *Immunity*.

[63] Dinarello C. A. (2009). Immunological and inflammatory functions of the interleukin-1 family. *Annual Review of Immunology*.

[64] Chen Z., O’Shea J. J. (2008). Th17 cells: a new fate for differentiating helper T cells. *Immunologic Research*.

[65] Dinarello C. A. (1999). IL-18: A TH1 -inducing, proinflammatory cytokine and new member of the IL-1 family. *The Journal of Allergy and Clinical Immunology*.

[66] Wang X., Feuerstein G. Z., Gu J. L., Lysko P. G., Yue T. L. (1995). Interleukin-1*β* induces expression of adhesion molecules in human vascular smooth muscle cells and enhances adhesion of leukocytes to smooth muscle cells. *Atherosclerosis*.

[67] Sola-Villa D., Camacho M., Sola R., Soler M., Diaz J. M., Vila L. (2006). IL-1*β* induces VEGF, independently of PGE2 induction, mainly through the PI3-K/mTOR pathway in renal mesangial cells. *Kidney International*.

[68] Mihai S., Codrici E., Popescu I. D. (2018). Inflammation-related mechanisms in chronic kidney disease prediction, progression, and outcome. *Journal of Immunology Research*.

[69] Vilaysane A., Chun J., Seamone M. E. (2010). The NLRP3 inflammasome promotes renal inflammation and contributes to CKD. *Journal of the American Society of Nephrology*.

[70] Allam R., Scherbaum C. R., Darisipudi M. N. (2012). Histones from dying renal cells aggravate kidney injury *via* TLR2 and TLR4. *Journal of the American Society of Nephrology*.

[71] Doi K., Ishizu T., Tsukamoto-Sumida M. (2014). The high-mobility group protein B1–Toll-like receptor 4 pathway contributes to the acute lung injury induced by bilateral nephrectomy. *Kidney International*.

[72] Allam R., Darisipudi M. N., Tschopp J., Anders H. J. (2013). Histones trigger sterile inflammation by activating the NLRP3 inflammasome. *European Journal of Immunology*.

[73] Tang T. T., Lv L. L., Pan M. M. (2018). Hydroxychloroquine attenuates renal ischemia/reperfusion injury by inhibiting cathepsin mediated NLRP3 inflammasome activation. *Cell Death & Disease*.

[74] Iyer S. S., Pulskens W. P., Sadler J. J. (2009). Necrotic cells trigger a sterile inflammatory response through the Nlrp3 inflammasome. *Proceedings of the National Academy of Sciences of the United States of America*.

[75] Shigeoka A. A., Mueller J. L., Kambo A. (2010). An inflammasome-independent role for epithelial-expressed Nlrp3 in renal ischemia-reperfusion injury. *The Journal of Immunology*.

[76] Gurung P., Anand P. K., Malireddi R. K. S. (2014). FADD and caspase-8 mediate priming and activation of the canonical and noncanonical Nlrp3 inflammasomes. *The Journal of Immunology*.

[77] Yabal M., Calleja D. J., Simpson D. S., Lawlor K. E. (2019). Stressing out the mitochondria: mechanistic insights into NLRP3 inflammasome activation. *Journal of Leukocyte Biology*.

[78] Orning P., Weng D., Starheim K. (2018). Pathogen blockade of TAK1 triggers caspase-8–dependent cleavage of gasdermin D and cell death. *Science*.

[79] Sarhan J., Liu B. C., Muendlein H. I. (2018). Caspase-8 induces cleavage of gasdermin D to elicit pyroptosis during *Yersinia* infection. *Proceedings of the National Academy of Sciences of the United States of America*.

[80] Gupta J., Mitra N., Kanetsky P. A. (2012). Association between albuminuria, kidney function, and inflammatory biomarker profile in CKD in CRIC. *Clinical Journal of the American Society of Nephrology*.

[81] Komada T., Chung H., Lau A. (2018). Macrophage uptake of necrotic cell DNA activates the AIM2 inflammasome to regulate a proinflammatory phenotype in CKD. *Journal of the American Society of Nephrology*.

[82] Chun J., Chung H., Wang X. (2016). NLRP3 localizes to the tubular epithelium in human kidney and correlates with outcome in IgA nephropathy. *Scientific Reports*.

[83] Yang S. M., Ka S. M., Hua K. F. (2013). Antroquinonol mitigates an accelerated and progressive IgA nephropathy model in mice by activating the Nrf2 pathway and inhibiting T cells and NLRP3 inflammasome. *Free Radical Biology & Medicine*.

[84] Hua K. F., Yang S. M., Kao T. Y. (2013). Osthole mitigates progressive IgA nephropathy by inhibiting reactive oxygen species generation and NF-*κ*B/NLRP3 pathway. *PLoS One*.

[85] Tsai Y. L., Hua K. F., Chen A. (2017). NLRP3 inflammasome: pathogenic role and potential therapeutic target for IgA nephropathy. *Scientific Reports*.

[86] Qiu Y. Y., Tang L. Q. (2016). Roles of the NLRP3 inflammasome in the pathogenesis of diabetic nephropathy. *Pharmacological Research*.

[87] Wada J., Makino H. (2016). Innate immunity in diabetes and diabetic nephropathy. *Nature Reviews Nephrology*.

[88] Wu M., Han W., Song S. (2018). NLRP3 deficiency ameliorates renal inflammation and fibrosis in diabetic mice. *Molecular and Cellular Endocrinology*.

[89] Shahzad K., Bock F., Dong W. (2015). Nlrp3-inflammasome activation in non-myeloid-derived cells aggravates diabetic nephropathy. *Kidney International*.

[90] Chen K., Zhang J., Zhang W. (2013). ATP-P2X4 signaling mediates NLRP3 inflammasome activation: a novel pathway of diabetic nephropathy. *The International Journal of Biochemistry & Cell Biology*.

[91] Garibotto G., Carta A., Picciotto D., Viazzi F., Verzola D. (2017). Toll-like receptor-4 signaling mediates inflammation and tissue injury in diabetic nephropathy. *Journal of Nephrology*.

[92] Solini A., Menini S., Rossi C. (2013). The purinergic 2X_7_ receptor participates in renal inflammation and injury induced by high‐fat diet: possible role of NLRP3 inflammasome activation. *The Journal of Pathology*.

[93] Goldwich A., Burkard M., Olke M. (2013). Podocytes are nonhematopoietic professional antigen-presenting cells. *Journal of the American Society of Nephrology*.

[94] Zhang C., Boini K. M., Xia M. (2012). Activation of Nod-like receptor protein 3 inflammasomes turns on podocyte injury and glomerular sclerosis in hyperhomocysteinemia. *Hypertension*.

[95] Gao P., Meng X. F., Su H. (2014). Thioredoxin-interacting protein mediates NALP3 inflammasome activation in podocytes during diabetic nephropathy. *Biochimica et Biophysica Acta (BBA) - Molecular Cell Research*.

[96] Feng H., Gu J., Gou F. (2016). High glucose and lipopolysaccharide prime NLRP3 inflammasome via ROS/TXNIP pathway in mesangial cells. *Journal of Diabetes Research*.

[97] Wang F., Huang L., Peng Z. Z. (2014). Losartan inhibits LPS + ATP-induced IL-1beta secretion from mouse primary macrophages by suppressing NALP3 inflammasome. *Die Pharmazie*.

[98] Yuan F., Kolb R., Pandey G. (2016). Involvement of the NLRC4-inflammasome in diabetic nephropathy. *PLoS One*.

[99] Moulton V. R., Suarez-Fueyo A., Meidan E., Li H., Mizui M., Tsokos G. C. (2017). Pathogenesis of human systemic lupus erythematosus: a cellular perspective. *Trends in Molecular Medicine*.

[100] Zhang W., Cai Y., Xu W., Yin Z., Gao X., Xiong S. (2013). AIM2 facilitates the apoptotic DNA-induced systemic lupus erythematosus via arbitrating macrophage functional maturation. *Journal of Clinical Immunology*.

[101] Manson J. J., Isenberg D. A. (2006). The origin and pathogenic consequences of anti-dsDNA antibodies in systemic lupus erythematosus. *Expert Review of Clinical Immunology*.

[102] Bai Y., Tong Y., Liu Y., Hu H. (2018). Self-dsDNA in the pathogenesis of systemic lupus erythematosus. *Clinical & Experimental Immunology*.

[103] Deshmukh U. S., Bagavant H., Fu S. M. (2006). Role of anti-DNA antibodies in the pathogenesis of lupus nephritis. *Autoimmunity Reviews*.

[104] Shin M. S., Kang Y., Lee N. (2013). Self double-stranded (ds)DNA induces IL-1*β* production from human monocytes by activating NLRP3 inflammasome in the presence of anti–dsDNA antibodies. *The Journal of Immunology*.

[105] Zhang H., Fu R., Guo C. (2016). Anti-dsDNA antibodies bind to TLR4 and activate NLRP3 inflammasome in lupus monocytes/macrophages. *Journal of Translational Medicine*.

[106] Kahlenberg J. M., Kaplan M. J. (2014). The inflammasome and lupus: another innate immune mechanism contributing to disease pathogenesis?. *Current Opinion in Rheumatology*.

[107] Li W., Titov A. A., Morel L. (2017). An update on lupus animal models. *Current Opinion in Rheumatology*.

[108] Meyer O. (1981). Animal models in lupus. *Revue du Rhumatisme et des Maladies Osteo-articulaires*.

[109] Waters S. T., Fu S. M., Gaskin F. (2001). NZM2328: a new mouse model of systemic lupus erythematosus with unique genetic susceptibility loci. *Clinical Immunology*.

[110] Zhao J., Wang H., Dai C. (2013). P2X_7_ blockade attenuates murine lupus nephritis by inhibiting activation of the NLRP3/ASC/caspase 1 pathway. *Arthritis & Rheumatism*.

[111] Fu R., Guo C., Wang S. (2017). Podocyte activation of NLRP3 inflammasomes contributes to the development of proteinuria in lupus nephritis. *Arthritis & Rheumatology*.

[112] Ka S. M., Lin J. C., Lin T. J. (2015). Citral alleviates an accelerated and severe lupus nephritis model by inhibiting the activation signal of NLRP3 inflammasome and enhancing Nrf2 activation. *Arthritis Research & Therapy*.

[113] Li X., Guo X., Liu H. (2017). Skin inflammation induced by lupus serum was inhibited in IL-1R deficient mice. *Clinical Immunology*.

[114] Lech M., Lorenz G., Kulkarni O. P. (2015). NLRP3 and ASC suppress lupus-like autoimmunity by driving the immunosuppressive effects of TGF-*β* receptor signalling. *Annals of the Rheumatic Diseases*.

[115] Manderson A. P., Botto M., Walport M. J. (2004). The role of complement in the development of systemic lupus erythematosus. *Annual Review of Immunology*.

[116] Benoit M. E., Clarke E. V., Morgado P., Fraser D. A., Tenner A. J. (2012). Complement protein C1q directs macrophage polarization and limits inflammasome activity during the uptake of apoptotic cells. *The Journal of Immunology*.

[117] Li J., Yan M., Zhang Y. (2017). Meta-analysis of the association between *NLRP1* polymorphisms and the susceptibility to vitiligo and associated autoimmune diseases. *Oncotarget*.

[118] Pontillo A., Reis E. C., Liphaus B. L., Silva C. A., Carneiro-Sampaio M. (2015). Inflammasome polymorphisms in juvenile systemic lupus erythematosus. *Autoimmunity*.

[119] Pontillo A., Girardelli M., Kamada A. J. (2012). Polimorphisms in inflammasome genes are involved in the predisposition to systemic lupus erythematosus. *Autoimmunity*.

[120] Wilson J. E., Petrucelli A. S., Chen L. (2015). Inflammasome-independent role of AIM2 in suppressing colon tumorigenesis via DNA-PK and Akt. *Nature Medicine*.

[121] Man S. M., Zhu Q., Zhu L. (2015). Critical role for the DNA sensor AIM2 in stem cell proliferation and cancer. *Cell*.

[122] Ponomareva L., Liu H., Duan X. (2013). AIM2, an IFN-inducible cytosolic DNA sensor, in the development of benign prostate hyperplasia and prostate cancer. *Molecular Cancer Research*.

[123] Dombrowski Y., Peric M., Koglin S. (2011). Cytosolic DNA triggers inflammasome activation in keratinocytes in psoriatic lesions. *Science Translational Medicine*.

[124] Choubey D., Panchanathan R. (2017). Absent in melanoma 2 proteins in SLE. *Clinical Immunology*.

[125] Panchanathan R., Duan X., Arumugam M., Shen H., Liu H., Choubey D. (2011). Cell type and gender-dependent differential regulation of the p202 and Aim2 proteins: implications for the regulation of innate immune responses in SLE. *Molecular Immunology*.

[126] Yang C. A., Huang S. T., Chiang B. L. (2015). Sex-dependent differential activation of *NLRP3* and *AIM2* inflammasomes in SLE macrophages. *Rheumatology*.

[127] Nakaya Y., Lilue J., Stavrou S., Moran E. A., Ross S. R. (2017). AIM2-like receptors positively and negatively regulate the interferon response induced by cytosolic DNA. *MBio*.

[128] Panchanathan R., Duan X., Shen H. (2010). *Aim2* deficiency stimulates the expression of IFN-inducible *Ifi202*, a lupus susceptibility murine gene within the *Nba2* autoimmune susceptibility locus. *The Journal of Immunology*.

[129] Mondini M., Vidali M., Andrea M. D. (2006). A novel autoantigen to differentiate limited cutaneous systemic sclerosis from diffuse cutaneous systemic sclerosis: the interferon-inducible gene IFI16. *Arthritis & Rheumatism*.

[130] Gugliesi F., Bawadekar M., De Andrea M. (2013). Nuclear DNA sensor IFI16 as circulating protein in autoimmune diseases is a signal of damage that impairs endothelial cells through high-affinity membrane binding. *PLoS One*.

[131] Kimkong I., Avihingsanon Y., Hirankarn N. (2009). Expression profile of HIN200 in leukocytes and renal biopsy of SLE patients by real-time RT-PCR. *Lupus*.

[132] Fang R., Hara H., Sakai S. (2014). Type I interferon signaling regulates activation of the absent in melanoma 2 inflammasome during *Streptococcus pneumoniae* infection. *Infection and Immunity*.

[133] Panchanathan R., Liu H., Leung Y. K., Ho S. M., Choubey D. (2015). Bisphenol A (BPA) stimulates the interferon signaling and activates the inflammasome activity in myeloid cells. *Molecular and Cellular Endocrinology*.

[134] Newman Z. L., Sirianni N., Mawhinney C. (2011). Auranofin protects against anthrax lethal toxin-induced activation of the Nlrp1b inflammasome. *Antimicrobial Agents and Chemotherapy*.

[135] Juliana C., Fernandes-Alnemri T., Wu J. (2010). Anti-inflammatory compounds parthenolide and Bay 11-7082 are direct inhibitors of the inflammasome. *Journal of Biological Chemistry*.

[136] Nuki G., Bresnihan B., Bear M. B., McCabe D., for the European Group Of Clinical Investigators (2002). Long-term safety and maintenance of clinical improvement following treatment with anakinra (recombinant human interleukin-1 receptor antagonist) in patients with rheumatoid arthritis: extension phase of a randomized, double-blind, placebo-controlled trial. *Arthritis & Rheumatism*.

[137] Lachmann H. J., Kone-Paut I., Kuemmerle-Deschner J. B. (2009). Use of canakinumab in the cryopyrin-associated periodic syndrome. *The New England Journal of Medicine*.

[138] Alten R., Gram H., Joosten L. A. (2008). The human anti-IL-1*β* monoclonal antibody ACZ885 is effective in joint inflammation models in mice and in a proof-of-concept study in patients with rheumatoid arthritis. *Arthritis Research & Therapy*.

[139] Perez-Gomez M. V., Sanchez-Nino M. D., Sanz A. B. (2015). Horizon 2020 in diabetic kidney disease: the clinical trial pipeline for add-on therapies on top of renin angiotensin system blockade. *Journal of Clinical Medicine*.

[140] Eser A., Colombel J. F., Rutgeerts P. (2015). Safety and efficacy of an oral inhibitor of the purinergic receptor P2X7 in adult patients with moderately to severely active Crohn’s disease: a randomized placebo-controlled, double-blind, phase IIa study. *Inflammatory Bowel Diseases*.

[141] Stock T. C., Bloom B. J., Wei N. (2012). Efficacy and safety of CE-224,535, an antagonist of P2X_7_ receptor, in treatment of patients with rheumatoid arthritis inadequately controlled by methotrexate. *The Journal of Rheumatology*.

[142] Keystone E. C., Wang M. M., Layton M., Hollis S., McInnes I. B., on behalf of the D1520C00001 Study Team (2012). Clinical evaluation of the efficacy of the P2X7 purinergic receptor antagonist AZD9056 on the signs and symptoms of rheumatoid arthritis in patients with active disease despite treatment with methotrexate or sulphasalazine. *Annals of the Rheumatic Diseases*.

[143] Kim H. J., Lee D. W., Ravichandran K. (2013). NLRP3 inflammasome knockout mice are protected against ischemic but not cisplatin-induced acute kidney injury. *The Journal of Pharmacology and Experimental Therapeutics*.

[144] Cao Y., Fei D., Chen M. (2015). Role of the nucleotide-binding domain-like receptor protein 3 inflammasome in acute kidney injury. *The FEBS Journal*.

[145] Arulkumaran N., Sixma M. L., Pollen S. (2018). P2X_7_ receptor antagonism ameliorates renal dysfunction in a rat model of sepsis. *Physiological Reports*.

[146] Gao P., He F. F., Tang H. (2015). NADPH oxidase-induced NALP3 inflammasome activation is driven by thioredoxin-interacting protein which contributes to podocyte injury in hyperglycemia. *Journal of Diabetes Research*.

[147] Liu Z., Wang X., Wang Y., Zhao M. (2017). NLRP3 inflammasome activation regulated by NF-*κ*B and DAPK contributed to paraquat-induced acute kidney injury. *Immunologic Research*.

[148] Gois P. H. F., Canale D., Volpini R. A. (2016). Allopurinol attenuates rhabdomyolysis-associated acute kidney injury: renal and muscular protection. *Free Radical Biology & Medicine*.

[149] Ka S. M., Kuoping Chao L., Lin J. C. (2016). A low toxicity synthetic cinnamaldehyde derivative ameliorates renal inflammation in mice by inhibiting NLRP3 inflammasome and its related signaling pathways. *Free Radical Biology & Medicine*.

[150] Niu X., Yao Q., Li W. (2019). Harmine mitigates LPS-induced acute kidney injury through inhibition of the TLR4-NF-*κ*B/NLRP3 inflammasome signalling pathway in mice. *European Journal of Pharmacology*.

[151] Wen Y., Pan M. M., Lv L. L. (2019). Artemisinin attenuates tubulointerstitial inflammation and fibrosis via the NF-*κ*B/NLRP3 pathway in rats with 5/6 subtotal nephrectomy. *Journal of Cellular Biochemistry*.

[152] Ding W., Xu C., Wang B., Zhang M. (2015). Rotenone attenuates renal injury in aldosterone-infused rats by inhibiting oxidative stress, mitochondrial dysfunction, and inflammasome activation. *Medical Science Monitor*.

[153] Anders H. J., Suarez-Alvarez B., Grigorescu M. (2018). The macrophage phenotype and inflammasome component NLRP3 contributes to nephrocalcinosis-related chronic kidney disease independent from IL-1-mediated tissue injury. *Kidney International*.

[154] Ludwig-Portugall I., Bartok E., Dhana E. (2016). An NLRP3-specific inflammasome inhibitor attenuates crystal-induced kidney fibrosis in mice. *Kidney International*.

[155] Song W., Wei L., Du Y., Wang Y., Jiang S. (2018). Protective effect of ginsenoside metabolite compound K against diabetic nephropathy by inhibiting NLRP3 inflammasome activation and NF-*κ*B/p38 signaling pathway in high-fat diet/streptozotocin-induced diabetic mice. *International Immunopharmacology*.

[156] Hsu W. H., Hua K. F., Tuan L. H. (2019). Compound K inhibits priming and mitochondria-associated activating signals of NLRP3 inflammasome in renal tubulointerstitial lesions. *Nephrology Dialysis Transplantation*.

[157] Iacobini C., Menini S., Blasetti Fantauzzi C. (2018). FL-926-16, a novel bioavailable carnosinase-resistant carnosine derivative, prevents onset and stops progression of diabetic nephropathy in *db/db* mice. *British Journal of Pharmacology*.

[158] Abais J. M., Xia M., Li G. (2014). Nod-like receptor protein 3 (NLRP3) inflammasome activation and podocyte injury via thioredoxin-interacting protein (TXNIP) during hyperhomocysteinemia. *Journal of Biological Chemistry*.

[159] Chang Y. P., Ka S. M., Hsu W. H. (2015). Resveratrol inhibits NLRP3 inflammasome activation by preserving mitochondrial integrity and augmenting autophagy. *Journal of Cellular Physiology*.

[160] Peng X., Yang T., Liu G., Liu H., Peng Y., He L. (2018). Piperine ameliorated lupus nephritis by targeting AMPK-mediated activation of NLRP3 inflammasome. *International Immunopharmacology*.

[161] Zhao J., Wang J., Zhou M., Li M., Li M., Tan H. (2019). Curcumin attenuates murine lupus via inhibiting NLRP3 inflammasome. *International Immunopharmacology*.

